# Carer and clinician perceptions of the use of emergency medical services by people with dementia: a qualitative study

**DOI:** 10.1017/S1463423618000191

**Published:** 2018-02-23

**Authors:** Sarah Voss, Janet Brandling, Sarah Black, Rik Cheston, Sarah Cullum, Steve Iliffe, Sarah Purdy, Jonathan Benger

**Affiliations:** 1 Associate Professor in Emergency and Critical Care, Faculty of Health and Life Sciences, University of the West of England, Bristol, UK; 2 Research Fellow, Faculty of Health and Life Sciences, University of the West of England, Bristol, UK; 3 Head of Research and Development, South Western Ambulance Service NHS Foundation Trust, UK; 4 Professor of Dementia Research, Faculty of Health and Life Sciences, University of the West of England, Bristol, UK; 5 Consultant in Old Age Psychiatry, University of Auckland, Auckland, New Zealand; 6 Professor of Primary Care for Older People, University College London, London, UK; 7 Head of Medical School, Faculty of Health Sciences, University of Bristol, Bristol, UK; 8 Professor of Emergency Care, Faculty of Health and Life Sciences, University of the West of England, Bristol, UK; 9 Consultant in Emergency Medicine, Emergency Department, University Hospitals Bristol NHS Foundation Trust, Bristol, UK

**Keywords:** ambulance service, dementia, emergency medical services

## Abstract

A growing number of older people are accessing emergency medical services (EMS), and many calls to EMS are made by, or on behalf of, people with dementia. Their needs are frequently complex; however, EMS staff are often given minimal guidance on ensuring patient safety, accurate diagnosis, and timely transfer to the most appropriate care. This study aimed to qualitatively explore the EMS experiences of carers for people with dementia and assess the views of EMS staff on the management of dementia, using focus groups and interviews. Themes were focussed on the circumstances surrounding EMS calls to people with dementia. These can prove frustrating due to a lack of information sharing, limited alternatives to hospital attendance and the amount of time that it can take to meet the complex needs of a person with dementia.

## Introduction

Whilst the majority of urgent care is delivered in primary care settings, an increasing number of older people are accessing emergency medical services (EMS) and emergency departments (EDs) (The Silver Book, 2012). It is estimated that 21–40% of older adults who present to the ED have cognitive impairment (Naughton *et al.*, 1995; Hustey and Meldon 2002). The oldest old are often frail; suffering from dementia, delirium or both, and if admitted for inpatient hospital care are three times more likely to die (EACSQHC, 2013). They also have the highest readmission rates, and highest rate of long-term care use after discharge (Sager *et al.*, 1996; Woodard *et al.*, 2010).

An average of nearly 2000 calls per day receive a face-to-face response from English EMS (NHS England, 2016). Just over 20% of older adults calling an emergency ambulance have dementia or cognitive impairment documented in their pre-hospital records (Buswell *et al.*, 2016); and it is likely that there is an additional group of patients with undiagnosed dementia. However, the use of EMS by older people with dementia is not well understood, and has been the subject of very little research. A review by Buswell *et al.* (2014) highlights the issue of ‘inappropriate’ calls, where an ambulance is called as the last resort or as a ‘safety net’.

Increasingly, EMS provides not just transport but a community response and treatment service, frequently discharging or referring older patients from the scene of a call, rather than conveying them to hospital. In order to deliver appropriate care for people with dementia, staff need continuing education about cognitive impairment in older adults. However, recent reviews have found there is minimal guidance for EMS staff (Clevenger *et al.*, 2012; Voss *et al.*, 2015; Buswell *et al.*, 2016).

This was an exploratory study to examine the experiences of EMS use by people with dementia from both clinical and carer perspectives. The aim of the study was to investigate the reasons why EMS calls are made, and the challenges faced by EMS clinicians when responding to people with dementia.

## Methods

This was a qualitative study with EMS staff and carers of people with dementia to investigate the experiences of EMS clinicians attending patients with dementia, and the experiences of carers of people with dementia when accessing English EMS.

EMS clinicians were employees of South Western Ambulance Service NHS Foundation Trust (SWASfT), and were recruited through the Trust bulletin. Seven paramedics, with an average of 7 years’ experience, participated in classic focus group discussions. These were facilitated by an independent qualitative researcher and held at SWASfT training premises.

The discussion topics were defined by the research team before the workshop and covered: recognising when a patient has cognitive impairment and consequential management; options for further interventions; conveyance to ED or referrals to primary care; discussion of a case vignette (Appendix 1). Group one was attended by three participants; one male and two females. Group two was attended by four participants; two males and two females. Each group lasted 2 h and was audio recorded.

Carers of people with dementia were recruited by advertising the study through Avon and Wiltshire Mental Health Partnership NHS Trust and their associated research and charity networks. Individual semi-structured telephone interviews (see Appendix 2 for interview schedule) were conducted with carers by an independent qualitative researcher to allow for exploration of individual reactions in the context of their own experiences.

Three interviews were conducted: A.A. is a carer for her mother who has advanced dementia and lives in her own home; A.G. cares for her mother who lives alone since the death of her husband; C.W. lives some considerable distance away from her father who has vascular dementia and lives alone. Interviews lasted between 23 and 42 min and were audio recorded.

Recordings from the focus groups and interviews were examined by the lead researcher (S.V.) and an independent qualitative researcher (J.B.) with notes taken and agreed. These notes were reviewed by the wider research team (authors), discussed and scrutinised for themes. A thematic approach from an interpretive paradigm (Braun and Clarke, 2006) was adopted to enable the identification, analysis, and reporting of patterns within data.

## Results

Data from the two population groups were analysed together. The circumstances surrounding an EMS call were divided into two broad themes: a carer calling EMS to avoid hospital, and a person with dementia making a call due to illness and/or confusion.

## Carer calling EMS

A.A. described a situation where an ambulance was called as there was no other option. A.A. felt her mother needed antibiotics but was unable to take her mother to the hospital by car. The general practitioner (GP) would not come out and suggested calling EMS.

However, the attending EMS clinician must consider all potential causes of cognitive impairment. If they are not able to rule out acute causes, such as head injury or infection which may need rapid treatment, they will convey the patient to hospital anyway.

### The carer perspective: ‘The last resort’

The EMS call is a last resort to access care, often motivated by desperation, even though they know it is not always an appropriate use of the service:

*Deep down I was aware that it kind of was taking up emergency services, probably. It wasn’t really what I would call a 999 emergency this was just a person with a specific need.*

(A.A.)

However, the reason for the call can be related to perceived hospital avoidance. There are occasions where the family believe the patient can be safely treated at home and they would like the EMS staff to initiate this, in the absence of other community options:

*She couldn’t go and wait at A&E for four hours and then get admitted.*

(A.A.)

*She ends up in hospitals for a couple of weeks and a couple of tests when actually there is no need for her to be there.*

(A.G.)

### The clinician perspective: ‘Need to make sure the patient is safe’

EMS staff must consider a variety of reasons for the presentation of cognitive impairment.

*Is there an infection going on that has the same kind of symptoms as cognitive impairment? Differentiating what’s what is really very difficult*.

(FG1, P1)

*We go to a lot of falls and it’s just not knowing whether the impairment is from the traumatic side or if it’s an ongoing issue and I think that’s something we probably come across a lot is not knowing the difference*.

(FG2, P1)

For clinicians, conveying the patient to hospital is sometimes the only safe option. C.W. described a situation where her father had been out walking and a passer-by had called an ambulance for him as they believed he had fallen. However, her father recalled he had just been sitting down for a rest. Patient safety then needs to be considered by EMS staff who have to be confident that there is no sign of head injury or other significant trauma in order to leave the patient at home or in the community.

… *and then the only place we can take them is A&E and that’s not the right place for them to go, you know*.

(FG1, P3)

*There are procedures that have to be followed… Not necessarily in the best interests of a dementia patient*.

(FG2, P4)

Both carers and clinicians expressed frustration at the lack of alternatives for people with dementia, with an ED visit becoming the default outcome.

*We all know that something needs to be done but the question is what? That’s something we encounter all the time*.

(FG2, P3)

These calls can take a lot of time, particularly when it is out of hours and there is a lack of engagement from other services:

*I was on a job for about 5 hours a while ago, social services wouldn’t come, the police wouldn’t come, I was being batted backwards and forwards*.

(FG2, P4).

*You could just be there for hours and hours because no-one is interested*.

(FG2, P2)

A similar scenario can develop when it is the person with dementia, rather than the carer, who calls the ambulance. This is illustrated in the second theme.

## Patient calling EMS

An EMS call is made by a person with dementia who is confused but not in need of urgent medical care. A.G. thinks that sometimes her mother feels unwell and calls 999 but her cognitive impairment means she does not always recall the reason by the time EMS clinicians arrive. Her mother may have described a headache due to a fall which happened several weeks ago, but the cognitive impairment means she is muddled about the cause of her headache. If there is no carer available and no other informant, the clinician may feel uncertain about leaving the person at home alone and will convey them to hospital.

### The carer perspective: patient is anxious or confused

Carers who do not live with their relative are aware that EMS calls are made but may not find out about it until after the event has happened. The person with dementia may make calls because of feelings of anxiety or confusion:

*Mum is phoning (999) instead of the GP*.

(A.G.)

*She’s confused and feels temporarily unwell so she calls but by the time they get there she has forgotten and she feels okay*.

(A.G.)

### The clinician perspective: need to make sure the patient is safe

EMS clinicians need to rule out urgent, time critical and life threatening conditions:

*With the acutely confused patient and there’s very little history you almost have to assume that it’s a stroke until proven otherwise*.

(FG1, P3)

*If you can’t speak to someone who knows them then you’ve got to assume the worst.*

(FG2, P4)

They may decide that conveying the patient to hospital on the basis of perceived medical and/or social need is a necessity:

*You just notice that the hob’s on, it’s been left on or something or the front door is open and then you think are they really safe to be left at home and yet there’s no real alternative*.

(FG2, P1)

*Your primary concern has got to be the safety of the patient. If you’re leaving them at home you’ve got to know they are safe*.

(FG2, P3)

EMS staff feel responsible for the safety of the patient and their decisions about whether to convey to hospital are influenced by both clinical and social circumstances.

## Discussion

The reasons why people with dementia access EMS are complex, and often related to the non-availability of other health services. EMS clinicians may be called by or for a person with dementia as a result of needing to access healthcare, but in the absence of more appropriate alternatives.

This gives rise to two significant problems. One is the arguably inappropriate use of urgent care services and resources, which prevents EMS clinicians from reaching incidents that require immediate and lifesaving resources. This is, in part, related to a lack of joined up services; EMS staff report spending large amounts of time at scene attending to the social needs of people with dementia due to a lack of alternative services.

The other problem relates to avoidable hospital admissions. These may occur when the attending EMS clinician is unable to rule out other causes of cognitive impairment such as sepsis or head injury, or when they feel it is unsafe to leave the patient at home for social reasons. In these instances, the patient is likely to be conveyed to the ED where they may be admitted unnecessarily. The ongoing ‘Keogh Review’ of Urgent and Emergency Care (Cummings, 2012), describes a system in which people with urgent but non-life threatening needs should be provided with effective services that deliver care in or close to their home. It is clear that there remains a gap between policy and reality, which needs to be addressed in order to meet long-term strategic health goals.

The findings from this study were limited by the small sample size and the fact that participant recruitment was limited to EMS staff and carers of people with dementia. Despite attempting to recruit through an NHS Trust and three associated charitable organisations, no people with dementia volunteered to participate. The reasons for this are unclear, but may reflect a lack of willingness to share experiences of EMS use, particularly if these were felt to be negative.

These findings help explain EMS use by people with dementia and give rise to important areas for further research. There is a need to gain a better understanding of EMS use by people with dementia. We know that EMS clinicians are called to these patients frequently, but there is no evidence to indicate the true burden of these calls, both in terms of the amount of time spent on scene and the cost of avoidable hospital admission.

## Conflicts of Interest

The authors declare no conflicts of interest.

## Ethical Standards

The study was approved by NRES Committee West Midlands (Ref: 14/WM/0127) and R&D approval was given by South Western Ambulance Service NHS Foundation Trust (Ref: 14-005) and Avon and Wiltshire Mental Health Partnership Trust (Ref: 854AWP).

## Financial Support

Financial support for the study was provided by University Hospitals Bristol NHS Foundation Trust (Research and Capability Funding: 16/2013-14).

## Appendix 1

**Mr and Mrs M**

You are called to a 73 year old man, Mr M, who has fallen in the kitchen at home. His wife is with him and has been unable to lift him from the floor. He has been on the floor for 90 minutes approximately. He has sustained bruising to his knee, elbow and shoulder and Mrs M reports that he did not hit his head and did not lose consciousness. He has been incontinent of urine, in moderate pain and is distressed.

Mr M is very well spoken and the house is a large, detached property in an affluent area. It is clean and well presented. He is dressed in a suit and tie but he is patchily shaven and there is some food spilled on his clothing. At first he is pleased to see you and grateful for assistance. He is a tall man and has fallen awkwardly against a cupboard and it takes a while to get in a position to lift him. He begins resisting your attempts to help him up and asks you to leave him alone. He gradually becomes more agitated, raising his voice and using his arms to deflect attempts to help. Mrs M tries to intervene to assist you and pacify her husband but he shouts at her using derogatory language and a scathing tone; Mrs M immediately retreats to the other side of the room.

Eventually, you are able to get Mr M off the floor and on to a chair. He is much calmer and tells you that he was a high ranking member of the army before he retired 10 years ago. He is enjoying retirement but mentions that he has recently resigned from being treasurer of the local bowls club as he felt the other club members don’t trust him. Mr M’s injuries are superficial and he does not require any further treatment.

A: You are happy that the fall was circumstantial and the injuries are mild.

*Would you leave the couple together at home? Would you take any further action?*

B: You are mildly concerned about Mr M’s agitated behaviour. You ask Mrs M if she has any concerns. Mrs M is vague but says that everything is perfectly okay and they are doing as expected for a couple in their 70’s. She says that neither of them have had to visit the GP recently for anything other than routine appointments.

*Would you leave the couple together at home? Would you take any further action?*

C: You notice that the garden is very unkempt in contrast with the interior of the house and that Mrs M has some evidence of yellowish bruising on her wrists. You take Mrs M through into another room and tell her that you noticed her husband seemed upset with her and ask if there is anything she needs. Mrs M says that her husband has been a little forgetful recently, but she thinks that is normal for people of their age. Upon further questioning, she reveals that he was asked to withdraw as treasurer of the bowls club as he was making a number of errors. She mentions that he is a proud man and prefers not to talk about any problems he faces but he is not performing some of the household duties that he used to do.

*Would you leave the couple together at home? Would you take any further action?*

D: You notice that the garden is very unkempt in contrast with the interior of the house and that Mrs M has some evidence of yellowish bruising on her wrists. You take Mrs M through into another room and tell her that you noticed her husband seemed upset with her and ask if there is anything she needs. Mrs M breaks down and tells you that her husband has been increasingly agitated and aggressive towards her over the past 12 months. He has been becoming very forgetful and she is worried about what this could mean; she thinks he should probably see a doctor but Mr M refuses to attend any appointment she makes. In any case, she is worried that the doctor may put him in a care home and as both their children live overseas, she would be alone. You ask further questions and Mrs M tells you that for the past 6 weeks, she has been performing many self-care tasks for her husband; washing and dressing him and assisting him with toileting. He is wakeful during the night and Mrs M is exhausted.

*Would you leave the couple together at home? Would you take any further action?*

## Appendix 2 - Interview Schedule

**Recognising and responding to cognitive impairment in emergency care: a pump priming project**

Pre-set question schedule: Patients and carers

1. What are your experiences of using emergency care, both positive and negative?
2. What do you believe to be the common issues in using emergency care and ambulance services for people with cognitive impairment?
3. Tell me about any experiences when you felt using emergency care and ambulance services was not a positive experience?
4. Tell me about any experiences when you felt using emergency care and ambulance services was a positive experience?

Pre-set question schedule: Staff

1. What are your experiences of attending patients with cognitive impairment, both positive and negative?
2. What do you believe to be the common issues for ambulance staff when they are attending people with cognitive impairment?
3. Tell me about any experiences when you felt attending a patient with cognitive impairment was not a positive experience?
4. Tell me about any experiences when you felt attending a patient with cognitive impairment was a positive experience?
